# Development and Assessment of Objective Surveillance Definitions for Nonventilator Hospital-Acquired Pneumonia

**DOI:** 10.1001/jamanetworkopen.2019.13674

**Published:** 2019-10-18

**Authors:** Wenjing Ji, Caroline McKenna, Aileen Ochoa, Haiyan Ramirez Batlle, Jessica Young, Zilu Zhang, Chanu Rhee, Roger Clark, Erica S. Shenoy, David Hooper, Michael Klompas

**Affiliations:** 1 Department of Pharmacy Administration and Clinical Pharmacy, School of Pharmacy, Xi’an Jiaotong University, Xi’an, Shaanxi, China; 2 Department of Population Medicine, Harvard Medical School and Harvard Pilgrim Health Care Institute, Boston, Massachusetts; 3 Department of Medicine, Brigham and Women’s Hospital, Boston, Massachusetts; 4 Department of Medicine, Brigham and Women’s Faulkner Hospital, Boston, Massachusetts; 5 Department of Medicine and Infection Control Unit, Massachusetts General Hospital, Boston

## Abstract

**Importance:**

Hospital-acquired pneumonia is the most common health care–associated infection in the United States. Most cases occur in nonventilated patients, but many hospitals track hospital-acquired pneumonia only in ventilated patients because of the complexity and subjectivity of conducting surveillance for large numbers of nonventilated patients.

**Objective:**

To propose and assess potentially objective, efficient, and reproducible surveillance definitions for nonventilator hospital-acquired pneumonia (NV-HAP) using routine clinical data stored in electronic health record systems.

**Design, Setting, and Participants:**

This cohort study was conducted in 2 tertiary referral and 2 community hospitals in Massachusetts between May 31, 2015, and July 1, 2018. All nonventilated patients aged 18 years or older who were admitted to these hospitals were included (N = 310 651).

**Exposures:**

Ten candidate definitions for NV-HAP based on clinically meaningful combinations of 6 potential surveillance criteria were proposed: worsening oxygenation, temperature higher than 38 °C (fever), abnormal white blood cell count of less than 4000/μL or more than 12 000/μL, performance of chest imaging, submission of respiratory specimen for culture, and 3 or more days of new antibiotics.

**Main Outcomes and Measures:**

Incidence rates, lengths of stay, hospital mortality rates, and odds ratios (ORs) for time to discharge and mortality compared with those of matched controls were calculated for each candidate definition. The ORs were adjusted for demographics, clinical service, comorbidities, and severity of illness.

**Results:**

The study analyzed 310 651 patients with 489 519 admissions, including 205 054 patients with 311 484 admissions of 3 or more days. Among the patients with 311 484 admissions, the mean (SD) patient age was 58.3 (19.3) years and 176 936 (56.8%) were of women. Incidence rates for candidate definitions per 100 admissions ranged from 3.4 events for worsening oxygenation alone to 0.9 event for worsening oxygenation and at least 3 days of new antibiotics to 0.6 event for worsening oxygenation, at least 3 days of new antibiotics, fever, abnormal white blood cell count, and performance of chest imaging. Crude mortality rates ranged from 16.1% (n = 2643) for patients with worsening oxygen alone to 27.7% (n = 868) for patients with worsening oxygenation, at least 3 days of antibiotics, fever or abnormal white blood cell count, and chest imaging. Patients who met NV-HAP candidate definitions remained in the hospital for twice as long as their matched controls (adjusted ORs ranged from 1.8 [95% CI, 1.7-1.8] to 2.1 [95% CI, 2.0-2.1]) and were 4 to 6 times as likely to die in the hospital (adjusted ORs ranged from 3.8 [95% CI, 3.5-4.0] to 6.5 [95% CI, 5.2-8.2]). Agreement between candidate definitions and clinical diagnoses was fair (κ = 0.33).

**Conclusions and Relevance:**

These findings suggest that objective surveillance for NV-HAP using electronically computable definitions that incorporate common clinical criteria is feasible and generates incidence, mortality, and adjusted ORs for hospital mortality similar to estimates from manual surveillance. These definitions have the potential to facilitate widespread, automated surveillance for NV-HAP and thus inform the development and evaluation of prevention programs.

## Key Points

**Question:**

Is it possible to conduct operational surveillance using the clinical data routinely recorded in electronic health records to identify nonventilated adults with hospital-acquired pneumonia?

**Findings:**

In this cohort study of 310 651 patients with 489 519 admissions, an electronic surveillance definition based on worsening oxygenation, at least 3 days of new antibiotics, fever or abnormal white blood cell count, and performance of chest imaging was successfully applied to all patients. This definition identified 0.6 event per 100 admissions and was associated with up to a 6-fold higher risk of hospital death compared with matched control patients.

**Meaning:**

This study suggests that electronic surveillance for nonventilator hospital-acquired pneumonia is feasible; this approach could inform the development and evaluation of pneumonia prevention programs in hospitals.

## Teaser

This cohort study develops and assesses electronic surveillance definitions for nonventilator hospital-acquired pneumonia using clinical data routinely recorded in electronic health records.

## Introduction

Hospital-acquired pneumonia is the most common health care–associated infection in the United States, affecting approximately 1% of hospitalized patients, two-thirds of whom are not receiving mechanical ventilation when pneumonia begins.^1,2,3^ Crude mortality rates for patients with nonventilator hospital-acquired pneumonia (NV-HAP) range between 15% and 30%, which are similar to or higher than the rates associated with ventilator-associated pneumonia.^3,4,5,6,7,8,9^ Most hospitals, however, do not have formal surveillance or prevention programs for NV-HAP.^3,10,11,12^

One of the major barriers to creating surveillance and prevention programs for NV-HAP is the complexity and subjectivity of the surveillance definitions published by the US Centers for Disease Control and Prevention (CDC).^13^ They require radiographic evidence of a “new or progressive and persistent infiltrate”; systemic signs, such as fever, abnormal white blood cell (WBC) count, or altered mental status; and pulmonary signs, such as rales, new cough, “new onset of purulent sputum, or change in character of sputum,” or “worsening gas exchange.”^13^^(p330)^ These criteria are subjective, nonspecific, and difficult to abstract because they are inconsistently documented and typically require reading free-text notes rather than analyzing structured data.^14,15,16^ The challenge is magnified by the size of the population at risk. In contrast to ventilator-associated pneumonia surveillance, almost every patient in the hospital must be tracked for NV-HAP rather than just the relatively small number of patients receiving mechanical ventilation. Furthermore, the correlation between clinical criteria and histological pneumonia is poor, and interobserver variability is high.^17,18,19^ Some researchers have proposed using administrative data for surveillance rather than clinical data, but diagnosis codes compound the limited accuracy of clinical criteria because they add coders’ interpretations to clinicians’ variable and imperfect impressions. The sensitivity of coding for NV-HAP is only between 40% and 60%, and the positive predictive value is between 30% and 50%.^3,20,21^

The limitations of current pneumonia definitions make routine surveillance for NV-HAP difficult for hospitals to conduct. This difficulty, in turn, limits hospitals’ capabilities to develop and evaluate pneumonia prevention programs. New surveillance definitions for NV-HAP are needed; ideally, these should be definitions that can be electronically applied using the data routinely found in electronic health record (EHR) systems. This would allow for the possibility of efficient, objective, and reproducible NV-HAP surveillance for large populations. Electronically computable definitions are unlikely to overcome the limited accuracy of clinical signs for pneumonia, but definitions that mirror clinical diagnostic criteria and that reliably and reproducibly detect nosocomial events associated with poor outcomes may provide a credible alternative to manual surveillance using the CDC traditional criteria.^13^

Given the potential value of an electronically computable surveillance definition for NV-HAP, we undertook a systematic evaluation of potential definitions using clinical data routinely found in most modern EHR systems. We identified potential clinical components to include in surveillance definitions; proposed candidate definitions using these components; and characterized the incidence, overlap with clinically diagnosed NV-HAP, and associations with time to discharge and hospital mortality for each candidate definition across 4 hospitals.

## Methods

### Study Design and Data Sources

We developed 10 candidate definitions for NV-HAP and then retrospectively applied them to all nonventilated patients aged 18 years or older who were admitted to 2 academic and 2 community hospitals in eastern Massachusetts (Brigham and Women’s Hospital, Massachusetts General Hospital, Newton-Wellesley Hospital, and Faulkner Hospital) between May 31, 2015, and July 1, 2018. Data for analysis were derived from the Epic EHR (Epic Systems Corp) using the Partners HealthCare Research Patient Data Repository, Enterprise Data Warehouse, and Microbiology databases. The study was reviewed and approved by the Partners HealthCare Institutional Review Board, which issued a waiver of informed consent because the study was retrospective, was observational, and presented results in aggregate. We followed the Strengthening the Reporting of Observational Studies in Epidemiology (STROBE) reporting guidelines.^31^

### Candidate Definitions

We identified clinical indicators routinely measured or obtained in pneumonia diagnosis that are (1) available for most patients in EHR systems, (2) objective, and (3) recorded as structured data. This yielded 6 possible clinical indicators: worsening oxygenation, new antibiotic administration, fever, abnormal WBC count, performance of chest imaging, and submission of respiratory specimens for culture. We did not include radiographic interpretations because they are subjective and nonspecific and require special expertise in natural language processing to parse that is not available in most hospitals.^19,22,23,24,25^ We also did not include arterial blood gases because they are measured in only a small subset of patients.

Next, we proposed 10 potential candidate definitions for NV-HAP surveillance that were based on clinically meaningful combinations of these 6 clinical indicators (Table 1). We required worsening oxygenation as an anchor sign for all candidate definitions because impaired oxygenation is a cardinal manifestation of pneumonia.^26,27,28,29^ All additional criteria included in each candidate definition (eg, fever, abnormal WBC count, and antibiotic starts) were required to be present on the first or second day of worsening oxygenation. Candidate definitions were modeled after the objective components of the CDC traditional surveillance criteria for hospital-acquired pneumonia but with adaptations similar to those implemented by the CDC to create ventilator-associated event definitions.^13,30^

**Table 1.** Candidate Definitions for Hospital-Acquired Pneumonia in Nonventilated Patients^a^

Candidate Definition	Worsening Oxygenation^b^	≥3 d of New Antibiotics^c^	Temperature >38 °C (Fever)	WBC Count <4000/μL or >12 000/μL	Chest Imaging Obtained	Respiratory Culture Obtained
1	✓	NA	NA	NA	NA	NA
2	✓	✓	NA	NA	NA	NA
3	✓	✓	✓^d^	✓^d^	NA	NA
4	✓	✓	✓	NA	NA	NA
5	✓	✓	✓	✓	NA	NA
6	✓	✓	✓	✓	✓	NA
7	✓	✓	✓^d^	✓^d^	✓	NA
8	✓	✓	✓	✓	✓	✓
9	✓	✓	✓^d^	✓^d^	✓^e^	✓^e^
10	✓	✓	✓	✓	✓^e^	✓^e^

Abbreviations: NA, not applicable; WBC, white blood cell.

SI conversion factor: To convert WBC count to ×10^9^ per liter, multiply by 0.001.

^a^ Check marks indicate that the variable is included in the candidate definition.

^b^ Worsening oxygenation was defined as at least 2 days of stable or improving oxygenation followed by at least 2 days of (1) decrease in daily minimum oxygen saturation from at least 95% in a patient on ambient air to less than 95% on ambient air, (2) initiation of supplemental oxygen, or (3) escalation of supplemental oxygen (eTable 1 in the Supplement). All additional criteria were required to be present on the first or second day of worsening oxygenation.

^c^ Less than 3 days of new antibiotics was allowed if the patient died on the first or second day of antibiotics.

^d^ Either temperature higher than 38 °C (fever) or WBC count less than 4000/μL or greater than 12 000/μL/

^e^ Either chest imaging obtained or respiratory culture obtained.

We defined *worsening oxygenation* as 2 or more days of stable or improving oxygenation followed by 2 or more days of any of the following: (1) a decrease in daily maximum oxygen saturation from 95% or higher in a patient on ambient air to less than 95% on ambient air, (2) the initiation of supplemental oxygen, (3) an increase in daily median oxygen flow rate of at least 3 L/min for a nasal cannula and at least 4 L/min for a simple face mask, or (4) an escalation in oxygen delivery device (nasal cannula → shovel mask → simple face mask → oxygen conservation device → high-flow oxygen → non-rebreather → noninvasive positive pressure ventilation → intubation and mechanical ventilation). The criteria for escalation of supplemental oxygen for different baseline states of oxygenation are further described in eTable 1 in the Supplement.

We defined *fever* as a daily maximum temperature higher than 38 °C and *abnormal WBC count* as more than 12 000/μL or less than 4000/μL (to convert to ×10^9^ per liter, multiply by 0.001). We counted both plain chest radiographs and computed tomography of the chest as eligible radiographic criteria. We defined *new antibiotic start* as antibiotics administered on hospital day 3 or later that were not given on the previous 2 calendar days. We required at least 3 days of new antibiotics to focus surveillance on the subset of potential pneumonias that clinicians deemed serious enough to merit sustained treatment. Antibiotics were limited to agents used to treat pneumonia (eTable 2 in the Supplement). New antibiotics started and continued for only 1 or 2 days were acceptable if the patient died before receiving a 3-day course. Antibiotic changes on the second or third day of treatment were permitted as long as there were no gaps of a calendar day or more in antibiotic administrations.

### Outcomes

We calculated incidence rates, time to discharge, and hospital mortality rates for each NV-HAP candidate definition. We included more than 1 potential NV-HAP event in a single admission for incidence estimates as long as the events were more than 14 days apart. However, we included only 1 randomly chosen event per admission to estimate the potential excess time to discharge and hospital mortality rate for each candidate definition. Incidence rates were expressed as the number of NV-HAP events per 100 admissions and per 1000 hospital days.

### Statistical Analysis

Data were analyzed from August 30, 2018, through August 23, 2019. We estimated potential excess time to discharge and hospital mortality rate by separately matching patients with each candidate definition with up to 4 randomly selected control patients. We required control patients to be drawn from the same hospital and clinical service as the case patients and to be hospitalized for at least as long as the time until the case patient’s second day of impaired oxygenation, which provided parity with our requirement for case patients to have at least 2 days of impaired oxygenation. Case patients were eligible to be controls for other cases as long as they were not yet cases as of the day of matching. We estimated the potential excess time to discharge using negative binomial regression as the rate ratio of the mean count of days from NV-HAP onset to hospital discharge across case patients divided by the mean count of days from match to hospital discharge across control patients. Potential excess mortality rate was estimated using logistic regression as the odds ratio (OR) of hospital death in cases compared with the controls.

We calculated crude and adjusted ORs with 95% CIs for all models. We adjusted for hospital, clinical service, age, sex, race/ethnicity, comorbidities (congestive heart failure, chronic lung disease, pulmonary vascular disease, diabetes, liver disease, kidney disease, neurological disease, stroke, lymphoma, and solid malignant neoplasm), Elixhauser Comorbidity Index,^32^ time to NV-HAP (match date), WBC count, hemoglobin level, hematocrit level, platelet count, serum creatinine, blood urea nitrogen level, sodium level, aspartate aminotransferase level, alanine aminotransferase level, total bilirubin level, albumin level, international normalized ratio, and history of antibiotic exposure between admission and day before meeting a given candidate definition. Missing laboratory values were imputed using the last known value or normal if never checked. We used robust SEs with generalized estimating equations to account for correlations within matched sets.^33^ Comparisons for each definition were estimated separately. All analyses were performed using SAS, version 9.4 (SAS Institute Inc).

High hospital mortality rates among patients who met NV-HAP candidate definitions may bias toward the null the excess time-to-discharge estimates among patients with NV-HAP. Therefore, we conducted a sensitivity analysis of time to discharge on survivors only. We also conducted sensitivity analyses including only patients with complete laboratory data and patients in whom respiratory cultures were obtained.

### Clinically Diagnosed NV-HAP

We evaluated the sensitivity, specificity, and positive predictive value of the combination of worsening oxygenation, new antibiotics, fever or abnormal WBC count, and chest imaging to identify clinically suspected NV-HAP through medical record reviews. We focused on this definition because it mirrors the existing CDC criteria for pneumonia and infection-related ventilator-associated complications.^13,30^ We randomly selected the medical records of 120 patients hospitalized for 3 or more days who met electronic surveillance criteria for impaired oxygenation. We nested the medical record reviews among patients with impaired oxygenation partly because of sampling efficiency, given the rarity of NV-HAP among undifferentiated patients, and partly because impaired oxygenation is a cardinal sign for pneumonia; the absence of impaired oxygenation makes pneumonia unlikely.^26,27,28,29^

One of us (H.R.B), who was blinded to the selection criteria, reviewed medical records for clinical documentation of suspected pneumonia (as defined by treating clinicians). Another one of us (M.K.) independently reviewed 10% of the medical records to confirm consistency and accuracy.

## Results

In total, 310 651 patients with 489 519 admissions were identified during the study period, including 205 054 patients with 311 484 admissions of 3 or more days. Among the patients with 311 484 admissions, the mean (SD) patient age was 58.3 (19.3) years and 176 936 (56.8%) were of women. The characteristics of all 311 484 admissions and patients who met a subset of candidate definitions are presented in Table 2. Data on the completeness of laboratory data and the success of matching are provided in eTable 3 in the Supplement.

**Table 2.** Patient Characteristics

Variable	All Patients Hospitalized for ≥3 d	Patients With Worsening Oxygenation	Worsening Oxygenation +≥3 d of New Antibiotics	Worsening Oxygenation +≥3 d of New Antibiotics + Fever or Abnormal WBC Count	Worsening Oxygenation +≥3 d of New Antibiotics + Fever or Abnormal WBC Count + Chest Imaging
Episodes, No.	311 484	16 466	4530	3627	3137
Age, mean (SD), y	58.3 (19.3)	64.3 (16.3)	64.5 (14.9)	63.9 (14.8)	63.7 (14.8)
Age group, No. (%)					
18-49	100 371 (32.2)	2817 (17.1)	656 (14.5)	553 (15.2)	484 (15.4)
50-64	79 077 (25.4)	4554 (27.7)	1356 (29.9)	1112 (30.7)	958 (30.5)
≥65	132 036 (42.4)	9095 (55.2)	2518 (55.6)	1962 (54.1)	1695 (54.0)
Male, No. (%)	134 548 (43.2)	8660 (52.6)	2667 (58.9)	2166 (59.7)	1931 (61.6)
Race/ethnicity, No. (%)					
White	239 815 (77.0)	13 561 (82.4)	3749 (82.8)	2976 (82.1)	2559 (81.6)
Black	27 191 (8.7)	1054 (6.4)	280 (6.2)	219 (5.9)	190 (6.1)
Hispanic	13 637 (4.4)	485 (3.0)	107 (2.4)	88 (2.4)	78 (2.5)
Asian	11 735 (3.8)	410 (2.5)	118 (2.6)	102 (2.8)	88 (2.8)
Other or missing	19 106 (6.1)	956 (5.8)	280 (6.2)	242 (6.7)	222 (7.1)
Clinical service, No. (%)					
Medicine	100 406 (32.2)	5063 (30.8)	1548 (34.2)	1203 (33.2)	831 (26.5)
Surgery	78 598 (25.2)	2228 (13.5)	552 (12.2)	409 (11.3)	312 (10.0)
Oncology	29 536 (9.5)	2630 (16.0)	841 (18.6)	696 (19.2)	550 (17.5)
Obstetrics	38 635 (12.4)	745 (4.5)	8 (0.2)	6 (0.2)	4 (0.1)
Cardiology	10 517 (3.4)	1089 (6.6)	228 (5.0)	180 (5.0)	169 (5.4)
Neurology	10 253 (3.3)	362 (2.2)	84 (1.9)	60 (1.7)	51 (1.6)
Cardiac surgery	3028 (1.0)	443 (2.7)	123 (2.7)	107 (3.0)	106 (3.4)
Gynecology	2925 (0.9)	69 (0.4)	19 (0.4)	12 (0.3)	10 (0.3)
Intensive care	26 685 (8.6)	3648 (22.2)	1063 (23.5)	906 (25.0)	831 (26.5)
Other or missing	10 091 (3.5)	189 (1.1)	64 (1.4)	48 (1.3)	39 (1.2)
Comorbidities, No. (%)					
Chronic lung disease	35 479 (11.4)	1841 (11.2)	415 (9.2)	315 (8.7)	248 (7.9)
Congestive heart failure	31 426 (10.1)	3141 (19.1)	896 (19.8)	722 (19.9)	659 (21.0)
Diabetes	46 904 (15.1)	3039 (18.5)	812 (18.0)	627 (17.3)	552 (17.5)
Renal failure	26 345 (8.5)	1688 (10.3)	422 (9.3)	310 (8.6)	270 (8.6)
Liver disease	12 003 (3.9)	710 (4.3)	238 (5.3)	187 (5.2)	167 (5.3)
Cerebrovascular disease	15 490 (5.0)	1162 (7.1)	360 (8.0)	297 (8.2)	266 (8.5)
Other neurological disorders	30 114 (9.7)	1866 (11.3)	510 (11.3)	386 (10.6)	330 (10.5)
Solid malignant neoplasms	23 198 (7.5)	1382 (8.4)	366 (8.1)	278 (7.7)	239 (7.6)
Cancer with metastases	22 130 (7.1)	1679 (10.2)	406 (9.0)	312 (8.6)	245 (7.8)
Lymphoma	9207 (3.0)	616 (3.7)	210 (4.6)	181 (5.0)	146 (4.7)
Elixhauser Comorbidity Index, mean (SD)	4.5 (8.2)	9.5 (10.1)	10.8 (10.4)	11.1 (10.4)	11.4 (10.5)
Time to NV-HAP, d					
Mean (SD)	NA	8.8 (11.9)	9.8 (13.1)	10.3 (13.4)	10.7 (13.9)
Median (IQR)	NA	5 (3-9)	6 (3-11)	6 (3-12)	6 (4-12)
Time from NV-HAP to discharge, d					
Mean (SD)	NA	11.3 (13.9)	14.2 (15.8)	14.9 (16.1)	15.3 (16.6)
Median (IQR)	NA	7 (4-14)	9 (5-17)	10 (6-18)	10 (6-19)
Hospital LOS, d					
Mean (SD)	7.1 (7.4)	20.1 (20.6)	24.0 (22.7)	25.2 (22.8)	25.9 (23.4)
Median (IQR)	5 (4-8)	14 (9-24)	17 (11-29)	18 (12-31)	19 (12-31)
Disposition, No. (%)					
Home	244 836 (78.6)	7448 (45.2)	1664 (36.7)	1267 (34.9)	1055 (33.6)
Rehabilitation facility	14 377 (4.6)	1589 (9.7)	423 (9.3)	335 (9.2)	285 (9.1)
Skilled nursing facility	36 963 (11.9)	4075 (24.8)	1191 (26.3)	939 (25.9)	818 (26.1)
Hospice	4143 (1.3)	618 (3.8)	149 (3.3)	121 (3.3)	96 (3.1)
Death	7475 (2.4)	2643 (16.1)	1081 (23.9)	945 (26.1)	868 (27.7)
Other	3690 (1.2)	65 (0.4)	14 (0.3)	13 (0.4)	9 (0.3)

Abbreviations: IQR, interquartile range; LOS, length of stay; NA, not applicable; NV-HAP, nonventilator hospital-acquired pneumonia; WBC, white blood cell.

The incidence of NV-HAP varied by candidate definition, ranging from 3.4 events per 100 admissions for patients with worsening oxygenation alone to 0.9 event per 100 admissions for patients with worsening oxygenation and at least 3 days of new antibiotics to 0.6 event per 100 admissions for patients with worsening oxygenation, at least 3 days of antibiotics, fever or abnormal WBC count, and performance of chest imaging (Table 3). Radiographic studies were obtained in 3795 patients (83.8%) with worsening oxygenation and antibiotics, but pulmonary cultures were obtained in only 1186 patients (26.2%) with worsening oxygenation and antibiotics. Given the low frequency of pulmonary cultures among patients who otherwise appeared to have possible NV-HAP, we focused on candidate definitions without this requirement.

**Table 3.** Incidence and Potentially Attributable Length of Stay and Mortality for Hospital-Acquired Pneumonia in Nonventilated Patients

Candidate Definition	Events Per 100 Admissions	Events Per 1000 Hospital Days	OR (95% CI)	
			Adjusted Time to Discharge	Adjusted Hospital Mortality
Worsening oxygenation	3.4	6.4	2.1 (2.0-2.1)	3.8 (3.5-4.0)
+ ≥3 d of new antibiotics	0.9	1.8	1.8 (1.7-1.9)	5.1 (4.6-5.8)
+ ≥3 d of new antibiotics + fever or abnormal WBC count	0.7	1.4	1.8 (1.7-1.9)	5.4 (4.8-6.1)
+ ≥3 d of new antibiotics + fever	0.3	0.6	1.8 (1.7-1.9)	5.0 (4.1-6.0)
+ ≥3 d of new antibiotics + fever + abnormal WBC count	0.2	0.5	1.8 (1.6-1.9)	5.9 (4.8-7.3)
+ ≥3 d of new antibiotics + fever + abnormal WBC count+ chest imaging	0.2	0.4	1.8 (1.7-1.8)	6.5 (5.2-8.2)
+ ≥3 d of new antibiotics + (fever or abnormal WBC count) + chest imaging	0.6	1.2	1.8 (1.7-1.9)	6.3 (5.5-7.2)
+ ≥3 d of new antibiotics + fever + abnormal WBC count+ chest imaging + respiratory culture	0.1	0.2	1.9 (1.7-2.2)	6.0 (4.2-8.7)
+ ≥3 d of new antibiotics + (fever or abnormal WBC count) + (chest imaging or respiratory culture)	0.6	1.2	1.8 (1.7-1.9)	5.8 (5.1-6.7)
+ ≥3 d of new antibiotics + fever + abnormal WBC count + (chest imaging or respiratory culture)	0.2	0.4	1.8 (1.7-1.9)	6.2 (5.0-7.8)

Abbreviations: OR, odds ratio; WBC, white blood cell.

The median (interquartile range [IQR]) hospital LOS among all patients admitted for 3 or more days was 5 (4-8) days. In contrast, the median (IQR) LOS for patients with NV-HAP ranged from 14 (9-24) days for the worsening oxygenation definition alone to 17 (11-29) days for worsening oxygenation and at least 3 days of new antibiotics to 19 (12-31) days for worsening oxygenation, at least 3 days of new antibiotics, fever or abnormal WBC count, and chest imaging. The adjusted ORs for time from NV-HAP to discharge in cases compared with controls ranged from 2.1 (95% CI, 2.0-2.1) for worsening oxygenation alone to 1.8 (95% CI, 1.7-1.8) for worsening oxygenation and at least 3 days of new antibiotics to 1.8 (95% CI, 1.7-1.9) for worsening oxygenation, at least 3 days of new antibiotics, fever or abnormal WBC count, and chest imaging. Sensitivity analyses limited to survivors yielded similar results (eTable 4 in the Supplement).

The crude hospital mortality rate among all patients hospitalized at least 3 days was 2.4% (n = 7475). In contrast, the crude mortality rates for patients who met candidate NV-HAP criteria ranged from 16.1% (n = 2643) for patients with worsening oxygenation alone to 23.9% (n = 1081) for patients with worsening oxygenation and at least 3 days of new antibiotics to 27.7% (n = 868) for patients with worsening oxygenation, at least 3 days of new antibiotics, fever or abnormal WBC count, and chest imaging. The adjusted ORs for hospital death in cases vs controls ranged from 3.8 (95% CI, 3.5-4.0) for patients with worsening oxygenation alone to 5.1 (95% CI, 4.6-5.8) for worsening oxygenation and at least 3 days of new antibiotics to 6.3 (95% CI, 5.5-7.2) for worsening oxygenation, at least 3 days of new antibiotics, fever or abnormal WBC count, and chest imaging, to 6.5 (95% CI, 5.2-8.2) for patients who met all the potential surveillance criteria. Sensitivity analyses restricted to patients with complete data and patients in whom pulmonary cultures were obtained generated similar results (eTable 5 in the Supplement).

Of the 120 medical records reviewed, 42 patients (35.0%) met the potential NV-HAP definition of worsening oxygenation, at least 3 days of new antibiotics, fever or abnormal WBC count, and chest imaging. Agreement between this candidate definition and clinicians’ working diagnoses was fair (κ = 0.33). Of the 42 patients flagged by this definition, treating clinicians documented concern for pneumonia in 25 patients (59.5%), with a positive predictive value of 60% (95% CI, 47%-71%). The potential reasons for deterioration in oxygenation for the remaining 17 patients (40.5%) included pulmonary edema, atelectasis, aspiration, pulmonary embolism, periprocedural airway management, and sepsis of nonpulmonary origin. Clinicians documented concern for pneumonia in 20 additional patients who did not meet the candidate electronic definition for NV-HAP, thus yielding a net sensitivity of 56% (95% CI, 40%-70%) and specificity of 77% (95% CI, 68%-86%).

Incidence rates varied widely between clinical services (Table 4). By the candidate definition of worsening oxygenation, at least 3 days of new antibiotics, fever or abnormal WBC count, and chest imaging, potential NV-HAP events per 100 admissions were most common in cardiac surgery (3.3 events), cardiology (1.2 events), oncology (1.7 events), and intensive care (2.9 events) units. Events were least common in the obstetrics (0 events) and gynecology (0.2 event) services. Incidence rates were higher in the 2 tertiary referral hospitals compared with the 2 community hospitals, but mortality rates were similar in all hospitals (Figure).

**Table 4.** Incidence of Selected Candidate Event Definitions by Clinical Service

Clinical Service	Events Per 100 Admissions			
	Worsening Oxygenation	Worsening Oxygenation + ≥3 d of New Antibiotics	Worsening Oxygenation + ≥3 d of New Antibiotics + Fever or Abnormal WBC **C**ount	Worsening Oxygenation + ≥3 d of New Antibiotics + Fever or Abnormal WBC **C**ount + Chest Imaging
Medicine	4.1	1.2	1.0	0.9
Surgery	2.0	0.5	0.4	0.3
Oncology	8.0	2.6	2.1	1.7
Obstetrics	1.7	0.0	0.0	0.0
Cardiology	7.6	1.6	1.3	1.2
Neurology	3.1	0.7	0.5	0.4
Cardiac surgery	14.0	3.9	3.4	3.3
Gynecology	1.3	0.3	0.2	0.2
Intensive care	12.6	3.7	3.1	12.9

Abbreviation: WBC, white blood cell.

**Figure.**
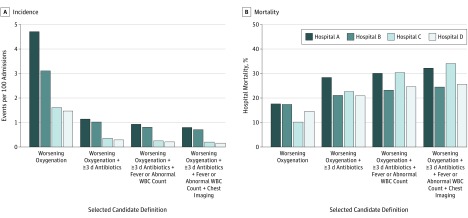
Comparative Incidence and Hospital Mortality Rates in Tertiary and Community Hospitals for Selected Candidate Definitions for Nonventilator Hospital-Acquired Pneumonia Hospitals A and B represent the tertiary hospitals, and hospitals C and D represent the community hospitals. WBC indicates white blood cell.

## Discussion

Nonventilator hospital-acquired pneumonia is among the most common health care–associated infections and is associated with high morbidity and mortality. Yet the complexity and subjectivity of traditional surveillance definitions have prevented most hospitals from implementing active surveillance and prevention programs for NV-HAP. This study demonstrated the feasibility of conducting surveillance using electronically computable surveillance definitions that mirror the clinical diagnosis and care patterns for NV-HAP, generate incidence rates similar to previously published manually generated estimates, and are associated with prolonged times to discharge and increased hospital mortality rates. These electronically computable events thus provide a potential means to support operational surveillance for NV-HAP and to inform the development of prevention and monitoring programs.

We proposed 10 candidate definitions for NV-HAP, ranging from worsening oxygenation alone to worsening oxygenation, fever, abnormal WBC count, performance of chest imaging, submission of pulmonary specimens for culture, and at least 3 days of new antibiotics. Incidence rates for each definition varied inversely with the number of surveillance criteria, with a sharp decrease in incidence for candidate definitions that required respiratory cultures. This decrease is consistent with previous observations that clinicians only obtain pulmonary specimens from a minority of patients with symptoms suggestive of pneumonia.^34,35^

A potentially credible surveillance definition, then, might be one that includes worsening oxygenation, at least 3 days of new antibiotics, fever or abnormal WBC count, and obtaining chest imaging. The incidence rate for this definition was 0.6 event per 100 admissions. This rate matches other investigators’ estimates of the prevalence of NV-HAP, including 2 multistate point-prevalence surveys conducted by the CDC that also estimated 0.6 event per 100 patients.^1,2,3,8,36,37^

Mortality rates for the candidate definitions mirrored the 15% to 30% mortality rates reported by other studies.^3,4,5,6,7,8^ We observed crude mortality rates ranging from 16.1% for worsening oxygenation alone to 27.7% for the combination of worsening oxygenation, at least 3 days of new antibiotics, fever or abnormal WBC count, and chest imaging. Similarly, patients who developed NV-HAP approximately doubled their time to discharge compared with patients who did not develop NV-HAP.

We found that the odds of hospital death for NV-HAP were high even after adjusting for a wide array of potential confounders, including hospital, clinical service, age, comorbidities, laboratory results, premorbid LOS, and previous antibiotic exposure. We estimated that patients with NV-HAP were 5 to 6 times more likely to die in the hospital compared with patients without NV-HAP. This finding mirrors the results from other studies. Micek and colleagues,^6^ for example, estimated an adjusted OR of death of 8.4 (95% CI, 5.6-12.5) for patients with NV-HAP.

Agreement between the proposed candidate definitions and clinical notations for pneumonia was only fair (κ = 0.33). Diagnosing pneumonia, however, is notoriously subjective and inaccurate.^17,18,19,20,21,38,39,40^ Many other studies have documented similarly low rates of interobserver agreement among infection preventionists (κ range = 0.12-0.40) and among clinicians (κ range = 0.16-0.34) who diagnose pneumonia.^14,15,16,40^ Furthermore, the positive predictive value of a clinical diagnosis of NV-HAP is only 30% to 50%.^3,20,21^ The significance of the mismatch between electronic surveillance criteria and clinical documentation is therefore unclear. Electronically computable definitions that use objective criteria may overcome some of the variability and inconsistencies inherent in traditional surveillance, but we have no reason to believe that they are any more (or less) accurate than traditional surveillance definitions.

Future studies are needed to prospectively validate the proposed surveillance definitions, further assess their correlation with clinical events labeled by physicians, identify risk factors, and measure their preventability by instituting best practices to prevent hospital-acquired pneumonia.

### Strengths and Limitations

The inclusion of antibiotic prescription criteria in the candidate definitions is both a strength and a weakness. It is a strength because it gives credence to clinicians’ impressions and limits detection to events that clinicians deemed serious and certain enough to warrant a sustained course of antibiotics. It is a weakness because clinicians vary in their predilection to diagnose and treat pneumonia, antibiotics may be administered for something other than pneumonia, overuse of antibiotics to treat possible pneumonia is common, and previous studies suggest that clinical impressions correlate poorly with biopsy or autopsy.^17,18,38,39,41^ The worsening oxygenation requirement, in contrast, likely adds some specificity to the definition, given that pulmonary impairment is a *sine qua non* for clinically meaningful pneumonia.^26,27,28,29^

This study has several limitations. First, it was conducted in 4 hospitals from a single region in the United States. Although the findings were similar across all study hospitals and matched independent estimates by other investigators, the results may differ in other clinical settings with distinct patient populations and clinical practice patterns. Second, the estimates of potential excess time to discharge and mortality rate may be biased by residual confounding, despite adjusting for a wide array of demographic and clinical factors. Third, we assessed only the association between surveillance definitions and clinically diagnosed pneumonia in patients with worsening oxygenation. This approach may have led to an overestimation of sensitivity. The accuracy and meaningfulness of clinically diagnosed pneumonia are questionable, however, in patients on ambient air with oxygen saturations consistently above 95%.^26,27,28,29^ Similarly, we reviewed only a small number of medical records, and thus the estimates of clinical correlates have wide confidence intervals. Fourth, the proposed candidate surveillance definitions are amenable for implementation only in hospital systems with comprehensive EHRs that include vital signs, demographics, antibiotic administrations, radiographic procedures, clinical cultures, and laboratory values as well as analytic support to extract and analyze these data. Disseminating this surveillance strategy will likely require sharing common data specifications and analytic code with hospitals and/or incorporating these definitions into commercial infection surveillance software systems.

## Conclusions

This study proposed objective surveillance definitions for NV-HAP that can be electronically computed using routine EHR data. These candidate definitions yielded incidence and mortality rates comparable to existing estimates based on manual surveillance. All definitions were associated with longer time to discharge and higher mortality rates. These definitions may therefore provide hospitals with an efficient and objective means to conduct routine surveillance for NV-HAP and thus to develop and evaluate prevention programs.

## Notes

Supplement. **eTable 1.** Criteria for Worsening Oxygenation **eTable 2.** Antibiotics Eligible for Inclusion in Candidate Definition Criteria **eTable 3.** Summary Data on Completeness of Laboratory Data and Success of Matching **eTable 4.** Sensitivity Analyses—Time to Discharge **eTable 5.** Sensitivity Analyses—Hospital Mortality
