# Supplementary material for: Development and Assessment of Objective Surveillance Definitions for Nonventilator Hospital-Acquired Pneumonia
Source: JAMA Netw Open. 2019 Oct 18;2(10):e1913674. doi: 10.1001/jamanetworkopen.2019.13674 (PMC6813588; doi:10.1001/jamanetworkopen.2019.13674)
Supplement: Supplement. — eTable 1. Criteria for Worsening Oxygenation eTable 2. Antibiotics Eligible for Inclusion in Candidate Definition Criteria eTable 3. Summary Data on Completeness of Laboratory Data and Success of Matching eTable 4. Sensitivity Analyses—Time to Discharge eTable 5. Sensitivity Analyses—Hospital Mortality [file jamanetwopen-2-e1913674-s001.pdf]

Supplementary Online Content

Ji W, McKenna C, Ochoa A, et al; CDC Prevention Epicenters Program. Development and assessment of objective surveillance definitions for nonventilator hospital-acquired pneumonia. *JAMA Netw Open*. 2019;2(10):e1913674. doi:10.1001/jamanetworkopen.2019.13674

eTable 1. Criteria for Worsening Oxygenation
eTable 2. Antibiotics Eligible for Inclusion in Candidate Definition Criteria
eTable 3. Summary Data on Completeness of Laboratory Data and Success of Matching
eTable 4. Sensitivity Analyses—Time to Discharge
eTable 5. Sensitivity Analyses—Hospital Mortality

This supplementary material has been provided by the authors to give readers additional information about their work.

© 2019 Ji W et al. *JAMA Network Open*

eTable 1. Criteria for Worsening Oxygenation

Baseline device/status \ New device/status	Ambient air & SpO_2 ≥95%	Ambient air & SpO_2 <95%	Nasal cannula	Simple face mask	Oxygen conserving device	Non-rebreather	High-flow oxygen	BIPAP	Ventilator
**Ambient air & SpO_2 ≥95%**		✓	✓	✓	✓	✓	✓	✓	✓
**Ambient air & SpO_2 <95%**			✓	✓	✓	✓	✓	✓	✓
**Nasal cannula**			↑3 lpm	✓	✓	✓	✓	✓	✓
**Simple face mask**				↑4 lpm	✓	✓	✓	✓	✓
**Oxygen conserving device**						✓	✓	✓	✓
**Non-rebreather mask**							✓	✓	✓
**High-flow oxygen**								✓	✓
**BIPAP**									✓

Abbreviations: lpm – liters per minute; SpO_2 – peripheral capillary oxygen saturation; BIPAP – bilevel positive airway pressure

© 2019 Ji W et al. *JAMA Network Open*

eTable 2. Antibiotics Eligible for Inclusion in Candidate Definition Criteria

Amikacin	Dalbavancin
Amoxicillin	Dicloxacillin
Ampicillin-sulbactam	Doxycycline
Azithromycin	Ertapenem
Aztreonam	Gentamicin
Cefadroxil	Imipenem
Cefazolin	Levofloxacin
Cefepime	Linezolid
Cefixime	Meropenem
Cefotaxime	Metronidazole
Cefotetan	Minocycline
Cefoxitin	Moxifloxacin
Cefpodoxime	Nafcillin
Ceftaroline	Oseltamivir
Ceftazidime	Oxacillin
Ceftazidime-avibactam	Penicillin
Ceftolozane-tazobactam	Peramivir
Ceftriaxone	Piperacillin-tazobactam
Cefuroxime	Polymyxin B
Cephalexin	Tedizolid
Ciprofloxacin	Tigecycline
Clarithromycin	Tobramycin
Clindamycin	Trimethoprim-sulfamethoxazole
Colistin	Vancomycin - IV

© 2019 Ji W et al. *JAMA Network Open*

eTable 3. Summary Data on Completeness of Laboratory Data and Success of Matching

Percent of events with incomplete data	Definition 1	Definition 2	Definition 3	Definition 4	Definition 5	Definition 6	Definition 7	Definition 8	Definition 9	Definition 10
WBC count	1.93%	0.60%	0.36%	0.54%	0.41%	0.44%	0.22%	0.52%	0.25%	0.43%
Hematocrit	1.90%	0.55%	0.30%	0.48%	0.33%	0.35%	0.19%	0.52%	0.22%	0.35%
Hemoglobin	1.91%	0.57%	0.30%	0.48%	0.33%	0.35%	0.19%	0.52%	0.22%	0.35%
Platelets	1.92%	0.55%	0.30%	0.48%	0.33%	0.35%	0.19%	0.52%	0.22%	0.35%
Serum sodium	4.64%	0.46%	0.25%	0.12%	0.08%	0.09%	0.13%	0.00%	0.16%	0.09%
Serum creatinine	4.50%	0.49%	0.28%	0.18%	0.08%	0.09%	0.13%	0.00%	0.16%	0.09%
ALT	37.37%	24.90%	22.61%	21.19%	19.62%	19.95%	21.71%	24.16%	21.63%	19.91%
AST	37.33%	24.88%	22.58%	21.13%	19.54%	19.86%	21.68%	23.90%	21.60%	19.83%
Total bilirubin	37.36%	24.59%	22.30%	20.82%	19.29%	19.60%	21.39%	23.38%	21.32%	19.57%
Albumin	35.92%	23.55%	21.26%	19.67%	18.56%	18.81%	20.34%	22.08%	20.25%	18.78%
INR	30.92%	21.17%	19.30%	21.37%	18.56%	17.85%	18.11%	18.96%	18.39%	18.17%
Any missing data	50.43%	36.71%	33.97%	34.50%	31.20%	30.53%	32.20%	31.95%	32.39%	30.87%
Number of matches per candidate NV-HAP event										
4	86.51%	96.28%	96.68%	97.88%	97.81%	98.03%	96.33%	97.35%	96.60%	98.13%
3	6.93%	1.30%	0.95%	0.44%	0.51%	0.45%	1.23%	0.53%	0.96%	0.36%
2	4.15%	1.26%	1.18%	0.31%	0.08%	0.18%	1.13%	0.00%	1.12%	0.09%
1	2.02%	0.67%	0.69%	0.56%	0.67%	0.36%	0.63%	0.80%	0.69%	0.45%
0	0.40%	0.49%	0.49%	0.81%	0.93%	0.99%	0.67%	1.33%	0.63%	0.98%

Abbreviations: ALT – alanine aminotransferase, AST – aspartate aminotransferase, INR – international normalized ratio, WBC – white blood cell

© 2019 Ji W et al. *JAMA Network Open*

eTable 4. Sensitivity Analyses—Time to Discharge

Candidate Definitions	Crude Days to Discharge OR (95% CI)	Adjusted Days to Discharge OR (95% CI)	Adjusted Days to Discharge Amongst Survivors Only OR (95% CI)	Adjusted Days to Discharge Amongst Patients with Complete Data OR (95% CI)	Adjusted Days to Discharge Amongst Patients in Whom Pulmonary Cultures Were Obtained OR (95% CI)
Worsening oxygenation	2.3 (2.3-2.4)	2.1 (2.0-2.1)	2.1 (2.1-2.1)	1.9 (1.9-2.0)	2.7 (2.6-2.9)
+ ≥3d antibiotics	1.9 (1.9-2.0)	1.8 (1.7-1.9)	1.9 (1.8-2.0)	1.7 (1.6-1.8)	2.2 (2.0-2.3)
+ ≥3d antibiotics + fever or leukocytosis	1.9 (1.9-2.0)	1.8 (1.7-1.9)	1.9 (1.8-2.0)	1.6 (1.5-1.7)	2.1 (1.9-2.2)
+ ≥3d antibiotics + fever	1.9 (1.8-2.1)	1.8 (1.7-1.9)	1.9 (1.8-2.1)	1.7 (1.6-1.9)	2.0 (1.9-2.2)
+ ≥3d antibiotics + fever + leukocytosis	1.9 (1.8-2.0)	1.8 (1.6-1.9)	2.0 (1.8-2.1)	1.6 (1.4-1.8)	2.0 (1.8-2.2)
+ ≥3d antibiotics + fever + leukocytosis + chest imaging	1.9 (1.7-2.1)	1.8 (1.7-1.9)	2.0 (1.8-2.2)	1.6 (1.5-1.8)	2.1 (1.9-2.3)
+ ≥3d antibiotics + (fever or leukocytosis) + chest imaging	2.0 (1.9-2.0)	1.8 (1.7-1.9)	2.0 (1.9-2.1)	1.6 (1.5-1.7)	2.1 (2.0-2.2)
+ ≥3d antibiotics + fever + leukocytosis + chest imaging + respiratory culture	2.0 (1.7-2.4)	1.9 (1.7-2.2)	2.4 (2.0-2.8)	1.8 (1.5-2.1)	1.9 (1.7-2.2)
+ ≥3d antibiotics + (fever or leukocytosis) + (chest imaging or respiratory culture)	2.0 (1.9-2.0)	1.8 (1.7-1.9)	2.0 (1.9-2.1)	1.7 (1.6-1.8)	2.1 (1.9-2.2)
+ ≥3d antibiotics + fever + leukocytosis + (chest imaging or respiratory culture)	1.9 (1.8-2.1)	1.8 (1.7-1.9)	2.1 (1.9-2.3)	1.6 (1.4-1.8)	2.0 (1.8-2.2)

© 2019 Ji W et al. *JAMA Network Open*

eTable 5. Sensitivity Analyses—Hospital Mortality

Candidate Definitions	Crude Hospital Mortality OR (95% CI)	Adjusted Hospital Mortality OR (95% CI)	Adjusted Hospital Morality Amongst Patients with Complete Data OR (95% CI)	Adjusted Hospital Mortality Amongst Patients in Whom Pulmonary Cultures Were Obtained OR (95% CI)
Worsening oxygenation	4.9 (4.6-5.2)	3.8 (3.5-4.0)	3.0 (2.7-3.5)	5.3 (4.4-6.4)
+ ≥3d antibiotics	6.2 (5.6-6.8)	5.1 (4.6-5.8)	3.9 (3.2-4.8)	7.2 (5.8-8.9)
+ ≥3d antibiotics + fever or leukocytosis	6.3 (5.7-7.0)	5.4 (4.8-6.1)	4.7 (3.8-5.8)	8.0 (6.5-9.9)
+ ≥3d antibiotics + fever	5.4 (4.7-6.3)	5.0 (4.1-6.0)	4.2 (3.1-5.7)	6.9 (5.2-9.3)
+ ≥3d antibiotics + fever + leukocytosis	6.3 (5.3-7.5)	5.9 (4.8-7.3)	5.2 (3.7-7.4)	5.8 (4.0-8.4)
+ ≥3d antibiotics + fever + leukocytosis + chest imaging	6.6 (5.5-7.9)	6.5 (5.2-8.2)	6.3 (4.5-8.8)	6.8 (4.8-9.7)
+ ≥3d antibiotics + (fever or leukocytosis) + chest imaging	7.0 (6.3-7.9)	6.3 (5.5-7.2)	5.4 (4.3-6.8)	7.9 (6.3-10.0)
+ ≥3d antibiotics + fever + leukocytosis + chest imaging + respiratory culture	7.1 (5.2-9.6)	6.0 (4.2-8.7)	7.3 (4.2-12.7)	6.0 (4.2-8.7)
+ ≥3d antibiotics + (fever or leukocytosis) + (chest imaging or respiratory culture)	6.6 (5.9-7.4)	5.8 (5.1-6.7)	4.9 (3.9-6.2)	6.9 (5.5-8.7)
+ ≥3d antibiotics + fever + leukocytosis + (chest imaging or respiratory culture)	6.4 (5.4-7.7)	6.2 (5.0-7.8)	4.8 (3.5-6.6)	7.3 (5.2-10.1)

© 2019 Ji W et al. *JAMA Network Open*
